# Optical coherence tomography and fractional flow reserve in below-the-knee percutaneous transluminal angioplasty: a pilot study

**DOI:** 10.1186/s42155-025-00580-9

**Published:** 2025-10-16

**Authors:** Chrissy van Wely, Rens J. Oosterveld, Lee H. Bouwman, Inge Fourneau, Arnoud W. J. van ‘t Hof, Ozan Yazar

**Affiliations:** 1 Department of Surgery, Division of Vascular and Endovascular Surgery, Zuyderland Medical Centre, Heerlen, the Netherlands; 2 Faculty of Science and Engineering, Maastricht University, Maastricht, the Netherlands; 3 Department of Vascular Surgery, University Hospitals Leuven, Leuven, Belgium; 4 Department of Cardiovascular Diseases, Leuven, KU Belgium; 5 Department of Cardiology, Maastricht University Medical Centre, Maastricht, the Netherlands; 6 Department of Cardiology, Zuyderland Medical Centre, Heerlen, the Netherlands; 7 Cardiovascular Research Institute Maastricht (CARIM), Maastricht, the Netherlands

## Abstract

**Purpose:**

Determining the safety of Optical Coherence Tomography (OCT) and Fractional Flow Reserve (FFR) in Percutaneous Transluminal Angioplasty (PTA) for below-the-knee vascular disease.

**Materials and methods:**

In this prospective single-center non-randomized trial, patients who underwent PTA for below-the-knee vascular disease with lesions no longer than 3 cm were included. Based on digital subtraction angiography (DSA) using iodine contrast agent, the physician was asked to estimate the diameter of the target vessel and degree of stenosis of the target lesion. The investigated tools are OCT, which is an intravascular imaging technique using near-infrared light and iodine contrast agent to visualize the vessel wall, and FFR, which measures the pressure gradient along a stenosis in a hyperemic state. The primary outcomes were the safety and feasibility of performing these measurements. OCT and FFR measurements were conducted before and after PTA. Physicians were not allowed to interpret study measurements during the procedure, as the safety and feasibility of these novel techniques have not yet been proven. To assess the secondary outcomes, physicians interpreted the measurements after the procedure to determine whether OCT or FFR would have changed intra-operative decision-making such as not performing PTA, the use of other balloons or stents, and additional revascularization based on the OCT and FFR measurements.

**Results:**

Ten patients were included. The target lesion was significant in nine patients based on angiography, who were therefore treated. Seven patients were treated with PTA using plain-old-balloon and two patients were treated using atherectomy devices.

No complications occurred during or after the procedures and measurements were successfully conducted in all patients. Overall, OCT and FFR would have led to a change in intra-operative decision-making in 7 patients. Estimation of the diameter of the target vessel varied from the value measured with OCT with more than 0.5 mm in 4 cases. FFR measurements demonstrated target lesions to be hemodynamically insignificant in 6 cases, while it showed target lesions to remain hemodynamically significant despite treatment in 3 cases.

**Conclusion:**

OCT and FFR are feasible to use in below-the-knee PTA and may cause significant alterations in perioperative decision-making by providing previously unavailable information on lesion size and morphology and hemodynamic significance.

## Introduction

Digital Subtraction Angiography (DSA) using iodine contrast is the golden standard imaging modality in Percutaneous Transluminal Angioplasty (PTA) of below-the-knee (BTK) arterial lesions [1]. This imaging modality can visualize the diameter of the target vessel, thus enabling the physician to estimate the degree of stenosis. However, this measurement is subjective and allows for intra- and interobserver variability. Therefore, DSA as a standalone tool does not allow precise measurement of the vessel diameter. This has been investigated in both crural and coronary arteries, as they are similar in diameter [2]. Secondly, DSA does not give the physician sufficient information on the morphology and shape of the plaque or stenosis in the vessel. Moreover, DSA cannot objectively determine whether a lesion causes significant loss of blood flow to the extremity [2]. These shortcomings make the outcome of the PTA dependent on the subjective assessment of the DSA made by the physician during the intervention.

Optical Coherence Tomography (OCT) can visualize the vessel wall from within the lumen and objectively measure its diameter and is used in this function in Percutaneous Coronary Intervention (PCI) [3, 4]. This allows for accurate selection of the correct balloon and stent size. Furthermore, OCT visualizes the morphology of the plaque and may visualize dissections in the vessel wall, the presence and extent of vessel wall calcifications and the presence of thrombus which may not be visible on DSA [3, 4]. OCT is superior compared to other intravascular imaging techniques due to its a tenfold larger resolution compared to other intravascular imaging techniques, such as intravascular ultrasound (IVUS), which allows for more precise measurement of vessel diameter, assessment of plaque morphology and in case of stent placement, assessment of stent apposition and expansion [4]. OCT has been proven to be of great value when determining the correct balloon or stent size and shows correct placement of the stent in terms of apposition and expansion, as used in PCI [3–6]. Fractional Flow Reserve (FFR) measures pre- and post-stenotic blood pressures both at rest and during a state of hyperemia, inducing decreased peripheral vascular resistance. FFR determines the hemodynamic significance of stenoses [4, 7]. FFR has reduced overtreatment of stenotic segments that would be assessed as significant based on DSA, but are in fact hemodynamically insignificant [4, 7].

Both OCT and FFR are techniques already in standard practice within the field of cardiology. They are regularly used in the coronary arteries during PCI and included in the European and American guidelines, demonstrating their significant value in intra-operative decision making [5, 6]. However, neither technique has been used or recommended in PTA for BTK arteries. In this pilot study we used OCT and FFR in ten patients undergoing PTA for below-the-knee lesions, to determine their feasibility and safety in treatment of below-the-knee arteries.

## Methods

### Subjects

**Fig. 1:**
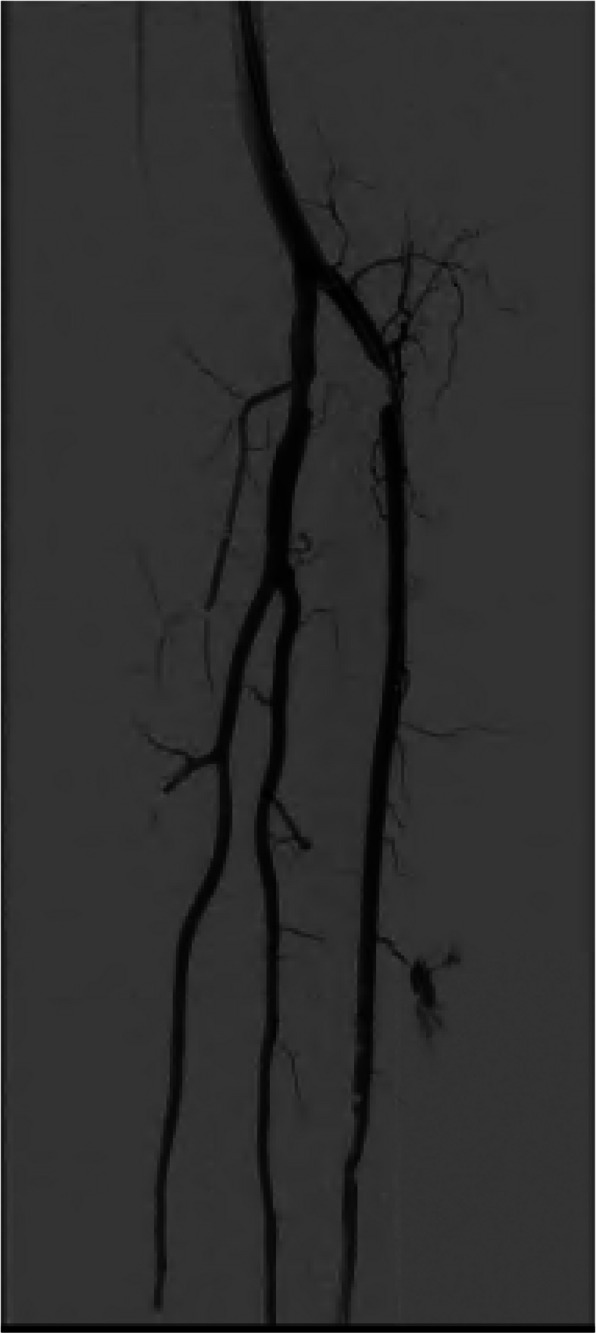
Example of a DSA with a crural lesion of < 3 cm. This angiography shows a short near occlusion in the proximal anterior tibial artery, with patent tibiofibular trunc, fibular artery and posterior tibial artery

Patients were eligible when the lesion on pre-operative imaging was no longer than 3 cm and located in below-the-knee arteries, classified according to the Global Anatomic Staging System Infrapopliteal as Grade 1. For an example of a DSA with such a target lesion see Fig. 1. Patients who underwent previous PTA at the index site within 30 days prior to the current intervention were excluded, as well as patients with previous major amputation on the ipsilateral leg and cases without informed consent. Demographic data was registered for all patients, including age, BMI, medical history, comorbidities, and vascular status.

### Procedure

The procedure started as per hospital protocol: patients received either general or local anesthesia, followed by an echo-guided antegrade puncture of the common femoral artery. Patients were given heparin based on the hospital protocol. A guidewire was introduced, followed by a sheath of the surgeons choosing. A DSA was made to visualize the femoropopliteal and crural arteries. Based on the initial DSA, the physician was asked to estimate the pre- and post-stenotic target vessel lumen, and the percentage of stenosis. As per the hospital protocol, this was done solely on the DSA without the use of additional measuring tools. Furthermore, the physician was asked whether treatment of the target lesion was indicated based on this DSA and which type and size balloon would be used.

After this, OCT and FFR measurements were conducted conforming to guidelines of the manufacturer as used in the coronary arteries. OCT measurements were made using the Dragonfly OPTIS™ Imaging Catheter combined with OPTIS™ Next Systems Software by Abbott Vascular (Santa Clara, CA, USA). An 0.014-inch guidewire was introduced, followed by an 8Fr sheath and a 6Fr guiding catheter, which was placed in the distal popliteal artery. The OCT catheter was introduced with its tip placed distal of the lesion. A bolus of approximately 16 ml iodine contrast agent was used to clear blood from the target vessel during the OCT pullback. FFR measurements were done using PressureWire X Guidewire by the same manufacturer, using papaverine to induce the hyperemic state. FFR measurement continued until a steady state was reached with a maximum of 90 s. The physician was not allowed to see or interpret these measurements during the procedure. After completing the measurements, PTA was conducted as planned based on the DSA. After confirming with DSA that the treatment was complete and the physician was satisfied with the result, OCT and FFR measurements were repeated. After completing the case, the physician was allowed to assess all OCT and FFR measurements of the patient and was asked whether the surgical procedure would have been carried out in a different manner, had the OCT and FFR been available during the procedure. As this is a proof-of-principle study that investigated the safety and feasibility of these novel techniques, the physician was not allowed to interpret the results during the procedure.

### Endpoints

The primary endpoint was completion of the crural PTA with OCT and FFR measurements without minor or major complications, similar to any catheterization procedure, such as dissection or occlusion, vessel perforation, embolization, or vessel spasm. Patients received regular follow-up including lab tests with Hb level, kidney function and infection parameters, and duplex-ultrasound 6 weeks after the procedure.

Secondary analysis was performed to investigate the manner in and extent to which OCT and FFR influence intra-operative decision-making. In this analysis is included the difference between vessel diameter and percentage of stenosis as estimated by the physician and as measured by OCT and FFR, the indication for treatment of the lesion as measured by FFR, the satisfactory completion of the treatment as measured by FFR, and complications seen with OCT that were not visible on post-operative DSA.

## Results

**Fig. 2:**
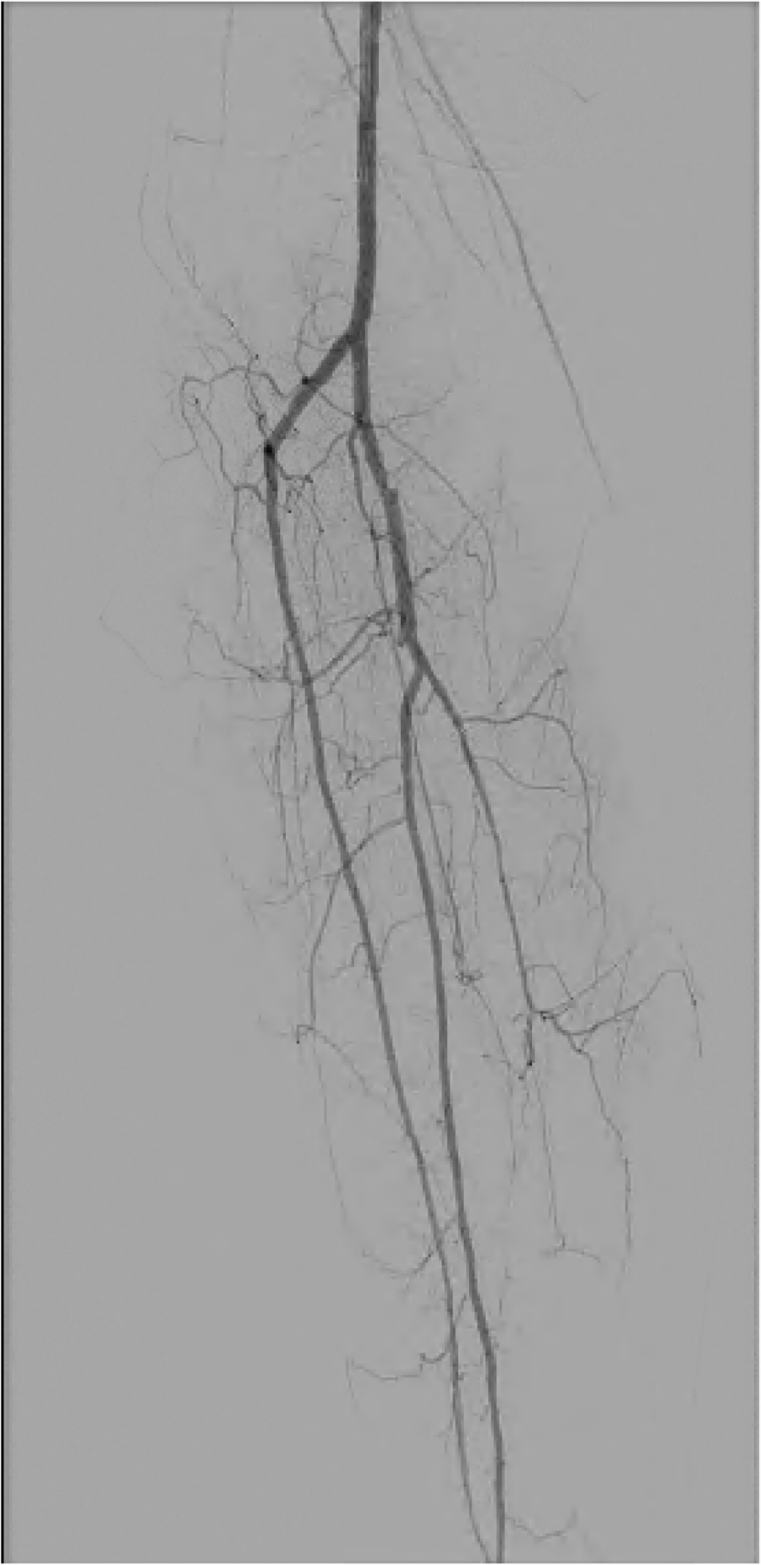
DSA of Patient 7, the lesion in the tibiofibular trunc was estimated as not significant. The anterior tibial artery and fibular artery were patent, the posterior tibial artery was occluding starting halfway the vessel. No revascularization was indicated

Ten patients were included in the analysis. Average age was 73 years old, and 90% was male. Patient demographics are found in Table 1. Indication for crural PTA was Rutherford class 4 or 5 vascular disease in all cases. Additional information on the vascular status and location of the wound can be found in Table 2. Based on DSA, PTA was indicated in 9 patients, whereas in 1 patient the physician decided no PTA was indicated due to the lesion being not significant on DSA (see Fig. 2). Jetstream atherectomy device was used in 2 patients followed by PTA with plain-old-balloon due to prior crural endovascular interventions resulting in restenosis at multiple levels. In all other cases, plain-old balloon was used for PTA. No stents were placed.

**Table 1:** Patient demographics. Mean age. History of vascular disease in the contralateral leg. Diabetes of any type. Hypertension defined as using medication to lower the blood pressure. Hypercholesterolemia defined as using cholesterol-lowering medication. History of Cerebro Vascular Attack (CVA) or Transient Ischemic Attack (TIA). History of coronary artery disease. History of smoking noted in the electronic patient file

**Factor**	**Number (% of total)**
Age	73
Male sex	8 (80%)
Contralateral vascular disease	3 (30%)
Diabetes	8 (80%)
Hypertension	8 (80%)
Hypercholesterolemia	10 (100%)
CVA/TIA	6 (60%)
Coronary artery disease	8 (80%)
History of smoking	6 (60%)

**Table 2:** Estimation of the diameter and degree of stenosis of the target vessel based on angiography. Diameter in millimeters and degree of stenosis in percentage. Measured diameter of the target vessel and degree of stenosis with OCT

**Patient**	**Estimation diameter ** **(mm)**	**Measured diameter** **(mm)**	**Estimated stenosis ** **(%)**	**Measured stenosis ** **(%)**
1	3	2,08	75	50
2	3	3,04	99	70
3	3	3,5	95	75
4	3	2,8	90	57
5	2	2,35	90	56
6	3,5	3,3	65	45
7	4	2,49	50	32
8	3	2,6	95	53
9	3	2,01	70	51
10	3	3,33	99	66

OCT and FFR measurements were successfully completed in all patients. No minor or major complications occurred during or after the procedure, caused by OCT or FFR techniques. Overall, intra-operative decision-making by the physician would have been changed by OCT or FFR in 7 patients (70%). The FFR values before and after treatment, the measurements and complications as seen on OCT, are summarized in Table 3.

**Table 3:** FFR values of the target lesions before and after balloon angioplasty. A lesion with an FFR value of 0.8 or lower is considered as hemodynamically significant

**Patient**	**FFR before treatment**	**FFR after treatment**	**Complication on OCT after treatment**
1	0,91	0,97	Dissections
2	0,52	0,61	
3	0,58	0,79	Dissections
4	0,89	0,99	trombus
5	0,58	0,61	
6	0,81	0,53	trombus
7	0,93	*	
8	0,98	0,9	Dissections
9	0,7	0,98	
10	0,84	0,84	Dissections

*Patient 7 was not treated with balloon angioplasty as the lesion was estimated to be hemodynamically insignificant. Therefore, no post-treatment FFR measurement was done. Complications as seen on OCT measurement after balloon angioplasty had finished

### Diameter and stenosis

Estimation of the diameter of the healthy lumen of the target vessel varied from the value measured with DSA compared to OCT by more than 0.5 mm in 4 cases (40%). Estimation of the degree of stenosis differed by more than 25% compared to the degree of stenosis measured by OCT in 6 cases (60%).

### Changes in perioperative decision-making after interpretation of FFR measurements

The physician decided that 9 patients required PTA, based on DSA. When using an FFR cut-off value of 0.8 for hemodynamic significance of a stenosis, FFR demonstrated the lesion to be hemodynamically insignificant in 6 cases (60%). The decision not to treat the lesion in one patient was confirmed by FFR and would not have changed the treatment decision. In the other 5 cases, FFR would have led to the physician not treating the target lesion.

**Fig. 3:**
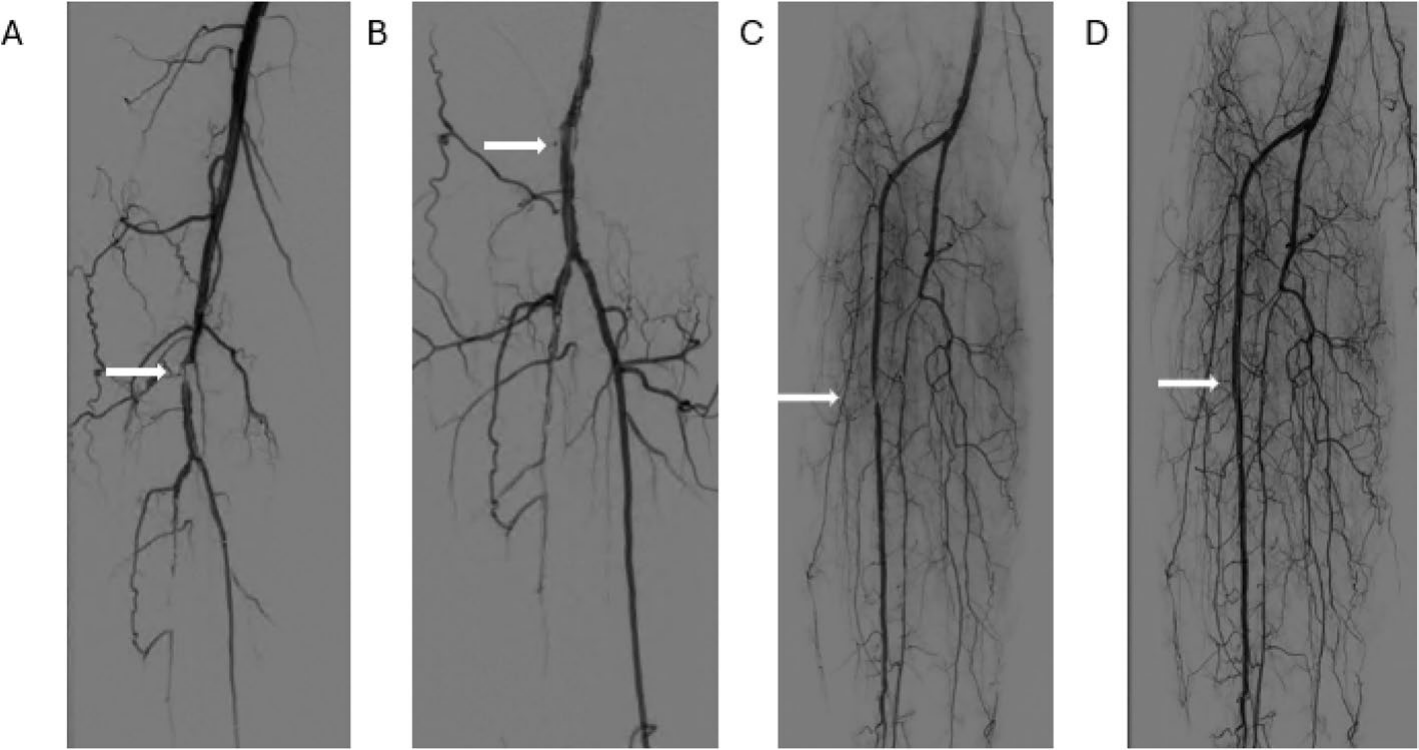
DSA of Patient 2 (**A** = pre-PTA, **B** = post-PTA) and Patient 5 (**C** = pre-PTA, **D** = post-PTA), demonstrating angiographic insignificant lesions indicated by the arrows after PTA that were hemodynamically significant based on the postoperative FFR measurement

Post-PTA FFR measurements demonstrated the target lesion to be persistently hemodynamically significant in 2 patients (20%), despite the physician being satisfied with the results based on DSA, demonstrated in Fig. 3. In these cases, the physician would have continued treatment based on the FFR value.

### Changes in perioperative decision-making after interpretation of OCT measurements

**Fig. 4:**
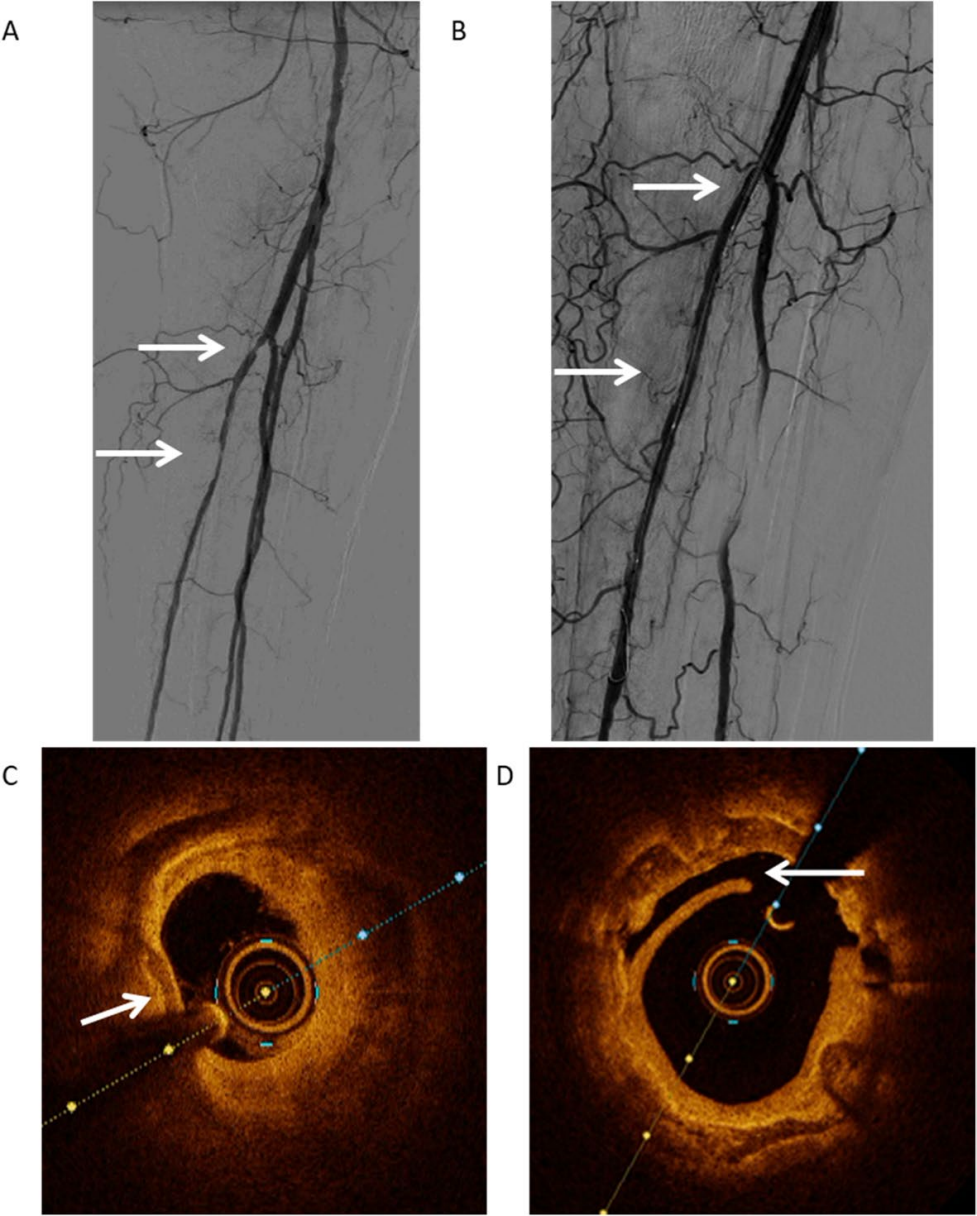
DSA and OCT images of Patient 1. Picture **A** shows the pre-intervention angiography of patient 1 with stenosis in the proximal posterior tibial artery. Picture **B** shows the post-intervention angiography of patient 1 without residual stenosis and no visible thrombosis or dissection. Picture **C** shows pre-intervention OCT image, demonstrating heavily calcified plaque indicated with the arrow with a severe loss of lumen. Picture **D** shows the post-intervention OCT of the same patient, demonstrating a widened lumen compared to the pre-intervention image with a dissection indicated with the arrow

**Fig. 5:**
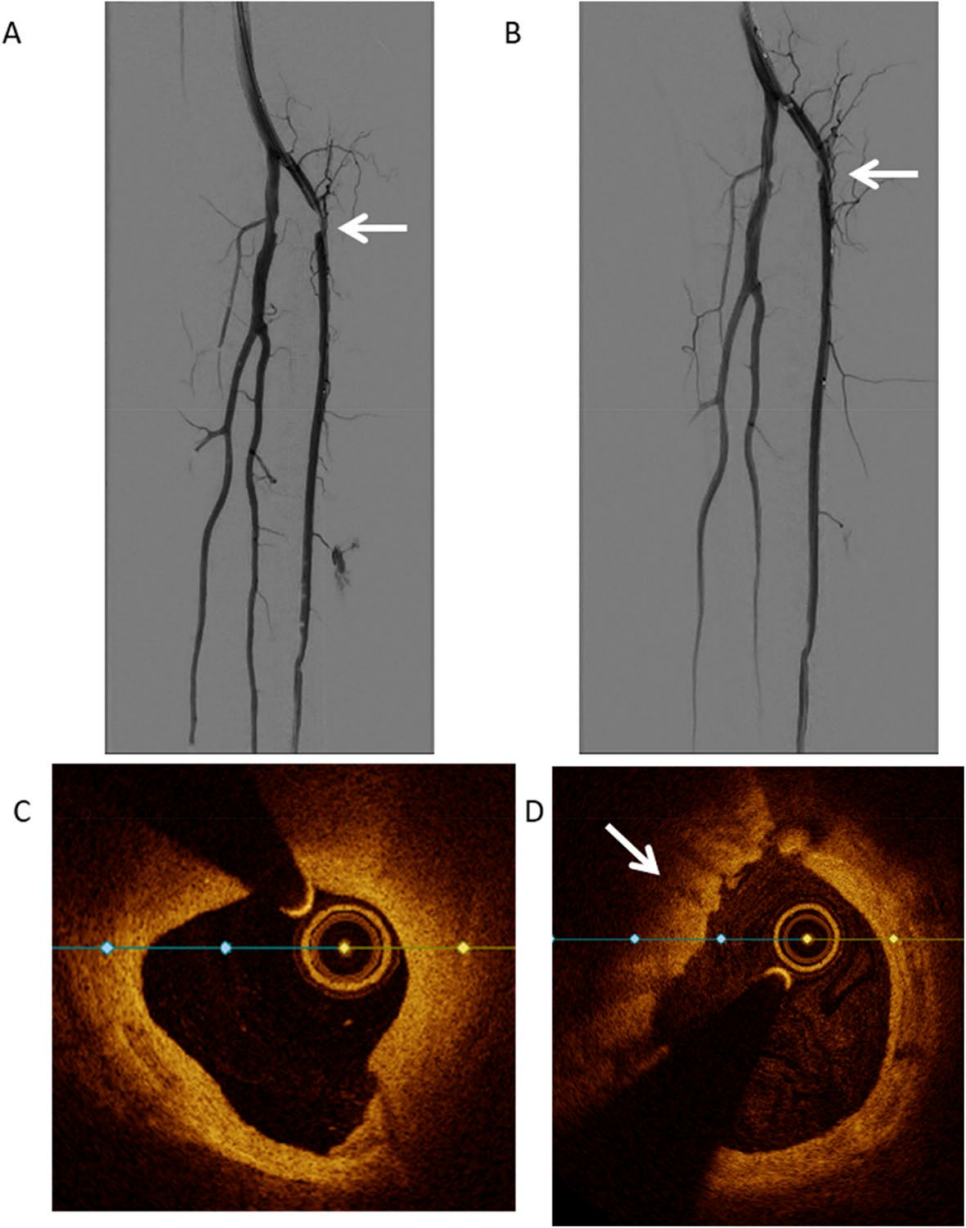
DSA and OCT images of Patient 4. Picture **A** shows the pre-intervention angiography of patient 4 with stenosis in the proximal anterior tibial artery. Picture **B** shows the post-intervention angiography with an improved lumen diameter and a non-flow compromising dissection and no clear thrombosis. Picture **C** shows pre-intervention OCT image demonstrating a loss of lumen due to calcified and lipid plaque. Picture **D** shows the post-intervention OCT of the same patient, demonstrating a widened lumen compared to the pre-intervention image with a fresh thrombus indicated with the arrow

OCT post-PTA demonstrated small dissections in 3 cases and major dissections in one case, despite the physician noting small non-flow limiting dissections on DSA in only 2 of these cases as demonstrated in Fig. 4. Furthermore, flow-limiting thrombus was seen in 2 patients, depicted in Fig. 5. Based on OCT, two cases would have received additional PTA with a different size balloon and stent placement.

## Discussion

This is a proof-of-principle study that used OCT and FFR in BTK PTA in 10 patients. Both techniques were safe to use and feasible, and measurements were conducted without complications. When used in clinical setting, the combination of OCT and FFR would have changed the intra-operative decision-making in seven out of ten patients.

In multiple patients, the combination of OCT and FFR would have led the physician to change the operative procedure. In several patients, FFR would have led the physician to decide not to treat a lesion because FFR determined the lesion to be hemodynamically insignificant. Ischemic pain and tissue loss in the foot may be caused by severely damaged microcirculation, causing outflow problems in the foot. Treating the target lesion in the proximal crural vessel may thus not solve the underlying problem. In several of these cases, treating the lesion led to dissections and thrombus that were not clearly visible on DSA but were visible on OCT. These dissections may cause early re-occlusion of the vessel, offering a possible explanation for the relatively low patency of crural PTA. Based on these findings, the physician may have wanted to continue treatment with balloon dilatation or stent placement. Further research needs to be done to identify which post-PTA dissections require further treatment, as not all dissections are clinically relevant.

The results in this pilot study correspond with previous articles describing the use of OCT in BTK PTA. Marmagkiolis et al. published a case-report using OCT in the popliteal artery of a limb ischemia patient. No perioperative complications occurred. Post-PTA OCT showed intraluminal protrusion of calcified plaque, which was not apparent on the DSA. Additional angioplasty was performed, and OCT showed optimal results [8]. Paraskevopoulos et al. reported the use of OCT to detect and characterize in-stent stenosis following infrapopliteal stent placement. OCT imaging was successfully completed in 19 out of 21 stents without major periprocedural complications, deeming it safe and feasible [9].

FFR would have changed intra-operative decision-making in 6 patients in our study. In several patients, FFR demonstrated the stenosis to be hemodynamically insignificant, while on DSA the stenosis was assessed as significant to treat. Furthermore, in 3 patients post-PTA FFR demonstrated the PTA to be unsuccessful since the lesion was still hemodynamically significant. Further research should be conducted on the prognostic value of FFR. We hypothesize that FFR could be used to predict clinical outcomes, as it is expected that lesions are not hemodynamically significant if the FFR is high. That would mean that treatment of the target lesion is not necessary, and local wound treatment should be optimalised. In case of non-healing wounds, amputation should be considered instead of numerous revascularization procedures. The FFR cut-off value as used in this study is the value used in cardiology guidelines, being 0.8 in hyperemic state. Although this value has been validated in cardiology, future research will need to determine the correct value for FFR in the lower leg. Within the current cardiology guidelines, FFR is used when in doubt of the hemodynamic significance of a lesion, reducing overtreatment of these lesions.

Previous research has shown comparable outcomes. Ruzsa et al. investigated the use of FFR in 39 chronic limb-threatening ischemia patients deeming it feasible and safe, however lacking sufficient data to draw conclusions on the cut-off value [10]. Limitations of the study by Ruzsa et al. include the lack of describing periprocedural complications or the influence of the value on perioperative decision-making.

In the current study, the influence of OCT and FFR on intra-operative decision making cannot be determined in detail due to the small study population. Although the principle and safety of the technique have been demonstrated in this study, further clinical trials with larger populations will have to demonstrate the value of adding OCT and FFR to DSA for crural PTA and determine the cut-off value for FFR in BTK lesions. As the additional measurements come with additional costs, it should also be taken into account that the costs of these novel techniques should be outweighed by the benefits. As we see that lesions may or may not have been treated based on the OCT or FFR measurements, it can be argued that we can prevent overtreatment of lesions by accurate anatomical and physiological measurements. This, however, needs to be investigated in larger clinical trials.

## Conclusion

OCT and FFR are safe and feasible to use in below-the-knee PTA and may cause significant alterations in perioperative decision-making by providing previously unavailable information on the hemodynamic significance of the lesion and its size and morphology.
